# Two weeks delayed bleeding in blunt liver injury: case report and review of the literature

**DOI:** 10.1186/1749-7922-6-14

**Published:** 2011-04-22

**Authors:** Uri Kaplan, Oshama A Hatoum, Alexander Chulsky, Hussam Menzal, Doron Kopelman

**Affiliations:** 1 Department of Surgery, Haemek Medical Center, Afula, Israel; 2 Faculty of Medicine, Technion - Israel Institute of Technology, Haifa, Israel; 3 Department of Radiology, Haemek Medical Center, Afula, Israel

## Abstract

Most cases of blunt hepatic trauma are treated nowadays non-operatively. This type of conservative treatment has resulted in increased complication rate. Delayed complications occur in cases that didn't require surgical intervention during the first 24 hours. The most common late complication is hemorrhage. We report a case of two weeks delayed hemorrhage after blunt hepatic trauma in an adult. We describe the diagnostic procedures, the surgical treatment and review the relevant literature.

## Case presentation

**Figure 1:**
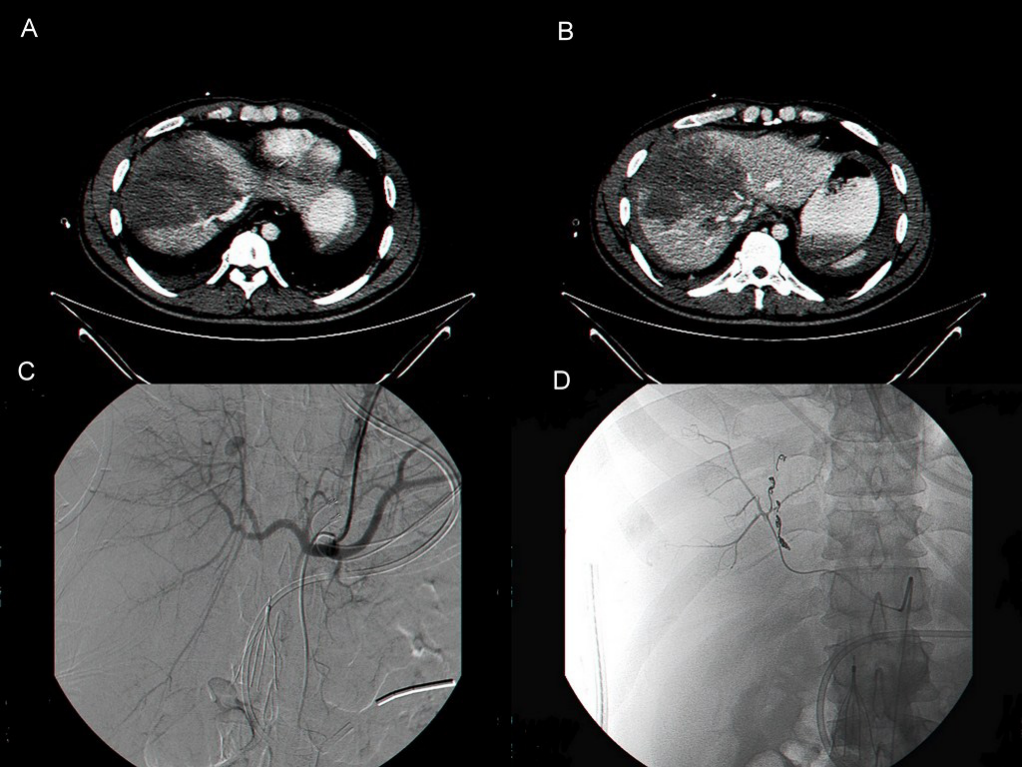
**A and B - CT scan on admission showing grade IV liver trauma; C- Angiogram showing pseudoaneurysm on the right liver; D- Angiogram after embolization with coils**.

A 40 year-old man was referred to our level II trauma center after a motorcycle road accident. On admission he was alert, without respiratory distress and complained of chest pain. Blood pressure (BP) was 124/58 mmHg and the pulse 85 beats/min. On examination, bruises were noted on his right thorax, and there was epigastric tenderness without signs of peritoneal irritation. Focused Assessment with Sonography for Trauma (FAST) revealed small amount of fluid in the pelvis. Chest and pelvic X-rays were normal. Being hemodynamically stable, computed tomography (CT) scans were performed. Chest CT showed minimal pneumothorax, fractured ribs 5 and 6, and minimal lung contusion on the right side. Abdominal CT showed a grade IV liver injury of the right lobe, accompanied by large amount of perihepatic fluid without evidence of active bleeding ("blush"), (Figure 1A, 1B). The patient, who required high doses of narcotics, was transferred to the intensive care unit (ICU) for sedation and close monitoring. At the ICU, A second CT scan revealed an increase in the amount of blood in the abdominal cavity with no active bleeding. He received 4 units of packed red blood cells (PC) and 2 units of fresh frozen plasma (FFP). Later, a large amount of right pleural transudate fluid was drained. Nine days after admission the severe pain subsided and he was transferred to the general surgery ward.

On the fifteen post trauma day, the patient suddenly complained of excruciating abdominal pain and became hemodynamically unstable. At that time his blood pressure was unmeasurable. The Hemoglobin level dropped from 10 g/dl to 7 g/dl. A short resuscitation enabled us to rush him to the operating room for an explorative laparotomy. Deep complex tears of the right liver lobe without active bleeding, but surrounded by fresh and old blood clots were found. The liver parenchyma was edematous, surprisingly soft and very fragile. Even a slight and otherwise minor maneuvering of the liver threatened to extend the damage. The clots were removed and due to the hemodynamical instability of the patient, packing around the liver was performed. Shortly after the operation, the patient's blood pressure dropped again and he was taken to angiography which didn't demonstrate signs of active bleeding. On that day the patient received 12 PC, 8 FFP and activated factor VII. Twenty four hours later, de-packing was performed, and the abdomen was temporarily closed with a Vac-pac dressing. During the first month the patient was confined to bed and was treated with intermittent compression device. Sixteen days after the trauma, and one day after his first surgery, an IVC filter was introduced. During the next 20 days the patient suffered from paralytic ileus, with extremely distended small bowel loops that prevented closure of the abdominal wall. Treatment with neostigmine facilitated bowel movements and two weeks later the abdominal wall was closed with a Vicryl mesh. On the 42^th ^hospitalization day, the patient developed again signs of hemodynamic instability, but his condition allowed an angiogram to be performed. Active bleeding from a pseudoaneurysm and an A-V fistula deep in the right lobe of the liver were detected. Bleeding was arrested by embolizing the vessel with coils (Figure 1C). On the 50^th ^day, once again the patient showed signs of instability. A third angiogram was performed and another pseudoaneurysm was detected and embolized with coils (Figure 1D). The patient remained hospitalized for another month. Three upper-abdominal abscesses were drained percutaneously under US guidance. The patient didn't have bile leaks. He had a few documented, clinically insignificant events of bacteremia during his stay in the ICU (contaminated cultures) and never suffered septic shock. He was mechanically ventilated from the day of his first surgery (day 15) until 33 days after his first trauma, 18 days in total. On the 83^rd ^post admission day, the abdominal wall was covered with skin grafts, and eight days later the patient was discharged and referred to a rehabilitation institute.

On follow-up six months later, he is well and asymptomatic with normal liver function tests. Permanent closure of the anterior abdominal wall is planned.

## Discussion

The treatment of blunt hepatic trauma has changed dramatically in the last two decades opting nonoperative management over operative treatment. The current rate of nonoperative treatment for blunt hepatic trauma being around 85-90% [1]. This change can be attributed to the improvement of the medical equipment: CT for the evaluation of the injury and angiography for the treatment of active bleeding. The published rate of successful nonoperative management of patients with isolated blunt liver injury is 91.5% for grade I and II, 79% for grade III, 72.8% for grade IV, and 62.6% for grade V injuries [2]. However, the resulting decline in the mortality rate was accompanied by a rise in the morbidity rate up to 7%. The most common complication of the nonoperative treatment is delayed hemorrhage that generally occurs in the first 72 hours [3-6]. The described case of sudden delayed bleeding fifteen days after the trauma is very rare. Due to the delay, such bleeding could have occurred after the patient's discharge from hospitalization. In our case, when the treatment strategy was decided upon, there was no sign of active vascular trauma. The patient was kept hospitalized that long despite his good physical status only because we wanted to perform another CT scan prior to discharge, which was delayed due to technical problems.

Delayed bleeding is treated either by angioembolization or surgically, depending on the hemodynamic condition of the patient. In our case, the hemodynamic instability required emergency laparotomy in the first event of delayed bleeding, but enabled us to use endovascular technologies in the recurrent two successive events. The mechanism of late bleeding in hepatic trauma is attributed to the clot breakdown into hyperosmolar fluid. More fluid is absorbed, increasing the size and pressure within the injured liver parenchyma until a breaking point is reached, tearing the tissue and causing bleeding. Such bleeding may either be sustained and form a pseudoaneurysm, create an arteriovenous fistula, or break into the peritoneal cavity. In the latter case, bleeding may be life threatening. Our patient developed all three possible types of late vascular complications.

The first event of active intraperitoneal bleeding occurred two weeks after the accident. A review of the literature revealed only one description of such a late bleeding in adults [7]. In this case the patient received 51 units of PC in order to deal with combined liver and spleen hemorrhage. In contrast to our case the patient, eventually, died. To our knowledge, there was no report of successful treatment after two weeks delayed bleeding from blunt liver trauma in adults and therefore our should be the first case to be published. Goettler et al. [8] published a case in 2002 describing delayed bleeding after blunt liver trauma in a pediatric patient. They reviewed the literature and found 11 such cases in children. The delay ranged from 8 hours to one month post trauma. The presentation included abdominal pain, hemodynamic instability and decreased hematocrit.

A significant resulting problem that we encountered was the handling of liver parenchyma during laparotomy. Usually, the trauma surgeon handles the liver parenchyma during laparotomy relatively early, within hours from the injury. At that time the consistency of the liver parenchyma is relatively normal. In our case, 15 days post trauma, we found a spongy, soft and very fragile liver parenchyma which was torn very easily and was difficult to handle. In consequence, we had to perform a damage control laparotomy only with packing of the liver. It appears that the first angiography performed shortly after this operation was prompted by a false alarm, as it did not detect any signs of active bleeding.

Kazar et al. [2] who reviewed the treatment of blunt liver trauma in adults, offered an algorithm that summarized the treatment. Based on the possible great delay in bleeding, we suggest that patients with complex blunt liver trauma (grades IV and V) who are managed nonoperatively, be followed by frequent US examinations, starting soon after the patient is stable. Such examinations may detect an increase in the size of the intrahepatic clots and parenchymal damage, indicating that a delayed bleeding may occur. Increased amounts of intraperitoneal fluid and suspicious changes in the liver texture should alert the surgeon and promote further imaging and angiographic studies. Such patients should be kept hospitalized to allow immediate surgery, should sudden massive intraperitoneal bleeding occur.

## Conclusion

Massive life threatening intraperitoneal bleeding may occur weeks after liver trauma. Although nonoperative management is opted nowadays over operative treatment, in high grades liver trauma, the patients should be closely monitored by US examinations to allow early detection of changes indicating the development of possible late complications. When such signs are detected, angiography may allow early nonoperative treatment and possibly prevent late bleeding. Patients should not be discharged before the pathological US imaging signs of damage are stabilized.

## Competing interests

The authors declare that they have no competing interests.

## Authors' contributions

All authors except AC were involved in the preoperative and postoperative care of the patient. UA is the primary author and reviewed the case and the literature. OAH participated in the surgeries and provided editorial commentary. AC performed the angiography treatment. DK performed the surgeries and was involved in the writing and editing the paper.

All authors have read and approved the final manuscript.

## Consent

Written informed consent was obtained from the patient for publication of this Case report and any accompanying images. A copy of the written consent is available for review by the Editor-in-Chief of this journal.
